# Sex‐dependent infection causes nonadditive effects on kissing bug fecundity

**DOI:** 10.1002/ece3.2956

**Published:** 2017-04-09

**Authors:** Carezza Botto‐Mahan, Verónica Campos, Rodrigo Medel

**Affiliations:** ^1^ Departamento de Ciencias EcológicasFacultad de CienciasUniversidad de ChileSantiagoChile; ^2^ Department of EntomologyPurdue UniversityWest LafayetteINUSA

**Keywords:** body size, host–parasite interaction, mating constraint, mating systems, size‐assortative mating

## Abstract

The influence of parasites on host reproduction has been widely studied in natural and experimental conditions. Most studies, however, have evaluated the parasite impact on female hosts only, neglecting the contribution of males for host reproduction. This omission is unfortunate as sex‐dependent infection may have important implications for host–parasite associations. Here, we evaluate for the first time the independent and nonindependent effects of gender infection on host reproductive success using the kissing bug *Mepraia spinolai* and the protozoan *Trypanosoma cruzi* as model system. We set up four crossing treatments including the following: (1) both genders infected, (2) both genders uninfected, (3) males infected—females uninfected, and (4) males uninfected—females infected, using fecundity measures as response variables. Interactive effects of infection between sexes were prevalent. Uninfected females produced more and heavier eggs when crossed with uninfected than infected males. Uninfected males, in turn, sired more eggs and nymphs when crossed with uninfected than infected females. Unexpectedly, infected males sired more nymphs when crossed with infected than uninfected females. These results can be explained by the effect of parasitism on host body size. As infection reduced size in both genders, infection on one sex only creates body size mismatches and mating constraints that are not present in pairs with the same infection status. Our results indicate the fitness impact of parasitism was contingent on the infection status of genders and mediated by body size. As the fecundity impact of parasitism cannot be estimated independently for each gender, inferences based only on female host infection run the risk of providing biased estimates of parasite‐mediated impact on host reproduction.

## 1 Introduction

The fitness impact of parasites ranges from early mortality and complete castration to slight reduction in host fecundity and even increased reproduction (Ballabeni, 1995; Minchella, 1985; Poulin, 1998). Even though reduced host fecundity is often an effect of parasitism, there is a still little knowledge about the mechanisms involved. Most existing evidence of parasite impact on host fitness in insects comes from studies performed on female hosts without consideration of the male infection status (Hurd, 2009). This omission is unfortunate considering that resource allocation trades off between parasite defense and other components of the phenotype differ between the sexes (Tschirren, Fitze, & Richner, 2003) resulting in sex‐specific strategies to avoid or tolerate parasitism. Thus, the infection status of males may affect their reproductive investment in different ways. For example, infected males may have lower energy allocation to spermatophore production, provide lower‐quality ejaculates, or fail to stimulate oviposition (Lehmann & Lehmann, 2000; Polak, 1996; Simmons, 1993). In these cases, females may receive direct benefits by mating with uninfected males. However, infected males may also positively affect the reproductive performance of females. For example, females lay more eggs when mated with infected males as infection increases the value of nuptial gifts (Hurd & Ardin, 2003). Therefore, the net resource availability for host reproduction may be contingent not only on female, but also on male infection status. Despite its importance, few studies have examined the influence of both male and female infections on host reproductive success (Sheridan, Poulin, Ward, & Zuk, 2000; Zuk & McKean, 1996), and to our knowledge, no study has examined potential interactive effects of sex‐dependent infection on host reproduction.

We focus on a host–parasite interaction between the hemipteran *Mepraia spinolai* (kissing bug) and the protozoan parasite *Trypanosoma cruzi*. Previous studies have reported that *T. cruzi* affects some life history traits of *M. spinolai*. For example, infected insects show longer developmental time and reduced body weight compared to uninfected insects (Botto‐Mahan, 2009). Likewise, gonads of infected females have 36.7% less weight than those of uninfected females (Botto‐Mahan, Ossa, & Medel, 2008). Here, we inquire into the importance of male and female infection costs on host reproduction using an experimental design that permits the assessment of additive (independent) and nonadditive (interactive) effects on host reproductive success. More specifically, if *T. cruzi* reduces both male and female host resource allocations to reproduction and effects are purely additive, the fecundity impact of parasitism can be estimated independently for each gender. On the contrary, under a nonadditive scenario, complex interactions between factors are expected. In such cases, the fecundity impact of infection on one sex cannot be estimated independently of the infection status of the other sex. Therefore, we address the following questions: (1) What are the effects, if any, of sex‐dependent infection on the reproductive success of *M. spinolai*? and (2) How important are nonadditive fecundity effects of parasitism in this system?

## 2 Materials and Methods

### 2.1 Study system

The kissing bug *M. spinolai* is a triatomine species responsible for *T. cruzi* transmission in mammals of arid and semiarid areas of Chile (Botto‐Mahan, Ortiz, Rozas, Cattan, & Solari, 2005). This strictly hematophagous and diurnal insect species is distributed between 26° and 34°S; its main habitat includes rocky outcrops, bird nests, rock crevices, and caves (Frías‐Lasserre, 2010). *Mepraia spinolai* requires the blood of vertebrates to complete its life cycle that includes egg, five nymph stages, and adult (Botto‐Mahan, 2009). In many triatomine species, one full engorgement is sufficient for molting from one stage to the next (Kollien & Schaub, 2000).

*Trypanosoma cruzi* is a heteroxenous trypanosomatid with a life cycle that involves several morphologically different stages, which can be found in insect vectors and mammalian hosts (Kollien & Schaub, 2000). This trypanosomatid multiplies and differentiates in the digestive tract of the insect vector. Infection of mammal hosts occurs by contamination of mucous membranes with insect feces, which contain the infectious metacyclic trypomastigote stage of the flagellate (Kollien & Schaub, 1997, 2000).

### 2.2 Infected and uninfected adults

Individuals of *M. spinolai* used in this study were obtained from the first generation of a cohort of field‐collected insects. During their development, insects (from the first instar nymph to adult) were reared individually in plastic containers maintained in a growth chamber at 26 ± 0.5°C, 65%–70% relative humidity, and 14 hr:10 hr light:dark cycle. Adults infected with *T. cruzi* were obtained by allowing insects to feed on infected laboratory rodents during their five nymphal stages. Only infected rodents in good condition and within the first 5 weeks of infection were used for feeding purposes (Wallace et al., 2001). Uninfected adults were obtained by allowing nymphs to feed on uninfected laboratory rodents.

The *T. cruzi* strain used in experimental infection was isolated from *M. spinolai* individuals collected in an endemic area, the same site where the parental kissing bugs were collected. Trypanosomes in feces of field‐captured insects were used to infect rodents by intraperitoneal inoculation. All individuals fed on infected rodents showed evidence of *T. cruzi* in their feces. Considering that previous reports indicate that *T. cruzi* often reduces kissing bug body size (Botto‐Mahan, 2009; Botto‐Mahan et al., 2008), adult body length was compared between infected and uninfected insects. All experiments were conducted with permission of the Ethical Committee of the Faculty of Science, University of Chile, and following the recommendations for animal testing (Goldberg, 2010).

### 2.3 Experimental design and reproductive output

Once reached the adult stage, combinations of infected and uninfected virgin insects were assigned to four mating treatments: infected males and females (*n *=* *19), infected males and uninfected females (*n *=* *16), uninfected males and infected females (*n *=* *25), and uninfected males and females (*n *=* *24). Each pair was in a 7‐cm height, × 6‐cm diameter plastic container with a meshed lid. Every container was provided with folded piece of paper as refuge. Laboratory conditions were as described above. All pairs fed to engorgement every 3 weeks on uninfected rodents. Pair survivorship and sexual activity (e.g., males mounting or trying to mount females) were recorded daily prior to removal of dead adults. While female adults were alive, eggs were collected from parental containers daily, counted, individually weighed (±0.05 mg), and placed in new containers. Eggs were classified as yolky or yolkless eggs considering deformations of surface and color. Eclosion of first instar nymphs was recorded daily until 1 month after the female parent's death.

### 2.4 Statistical methods

To examine the effect of the infection status of males, females, and their interaction, two‐way ANCOVAs were used. The infection status of males and females was the main factor. The total time spent together (i.e., total time the pair was together) and adult female survivorship (i.e., time elapsed from the first day of mating to female death) were the covariates. Dependent variables consisted of the following: (1) production of yolky eggs, (2) weight of yolky eggs, (3) production of yolkless eggs, (4) reproductive investment (number × mean weight of yolky eggs), (5) the day by which the female laid 50% of her eggs (E50, hereafter), and (6) the number of first‐stage nymphs. All dependent variables were checked for homogeneity of variance and normality and transformed when needed. For the number of nymphs, we used GLM with Poisson distribution errors and log link to compare treatments. When the interaction between female infection status and male infection status was significant, we examined the significance of each main effect by comparing one specific factor at a variable level of another using interaction slices (Schabenberger, Gregoire, & Kong, 2000). All analyses were performed in JMP version 8.0.2.

Because differences in reproductive output can be attributable not only to an effect of *T. cruzi* but also to variation in the volume of ingested blood, we compared the volume of blood ingested by females in a one‐way ANCOVA, using body size as covariate and female infection status as main effect. Body size was estimated as a factor from the linear combination of the equations for body length (mm), abdomen width (mm), and body weight (mg), which together accounted for 91.7% of the variance. In addition, we compared survivorship between infected and uninfected adult males and females, calculated as the number of days elapsed between the first day of mating and its death, using Student's *t* tests (Sokal & Rohlf, 1995).

## 3 Results

**Figure 1:**
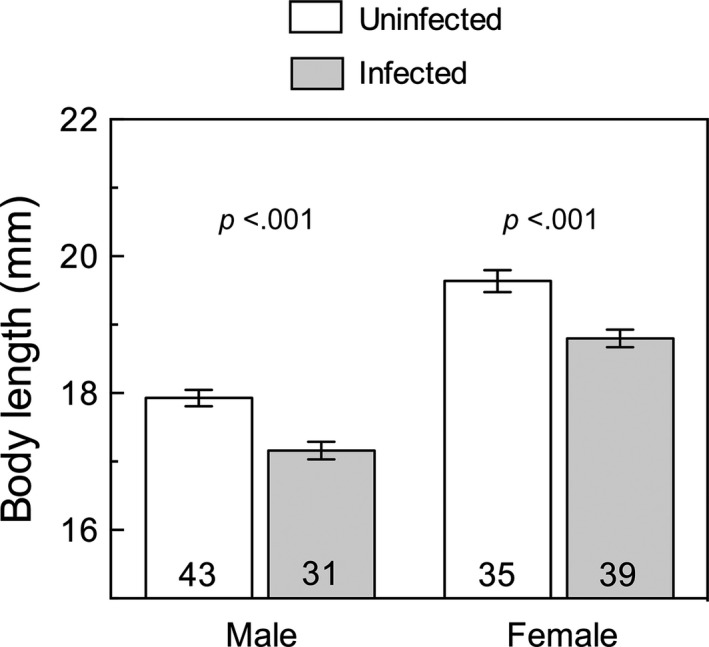
Mean (±1 *SE*) body length of uninfected (white bars) and infected (gray bars) males and females. Numbers at the bottom of bars indicate individual replicates. *p*‐Values correspond to the effect of infection status within each sex

**Figure 2:**
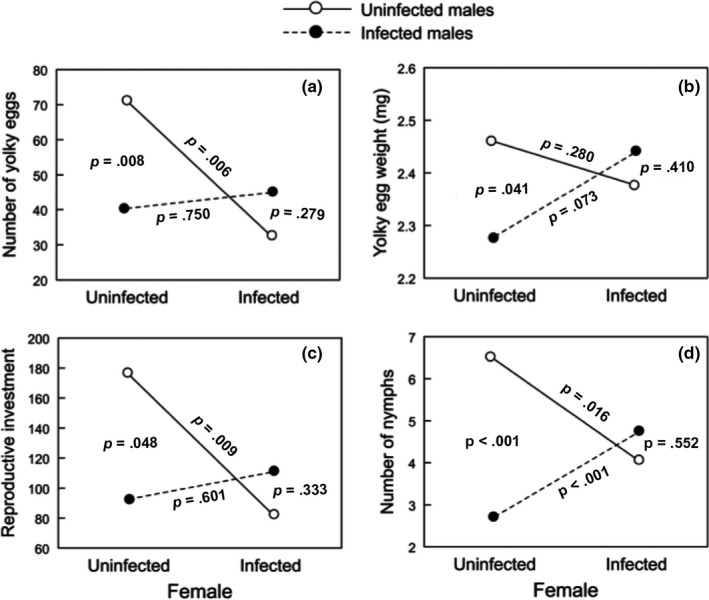
Interaction graphs for the effects of sex and infection status on the mean: the number of yolky eggs (a), weight of yolky eggs (b), reproductive investment (c), and the number of nymphs (d). Circles indicate model‐adjusted cell means with uninfected males (open circles and continuous lines) or infected males (filled circles and dashed lines). The values plotted are back‐transformations of the least squares means obtained from two‐way ANCOVAs on log‐transformed data. *p*‐Values indicate the statistical significance of the four effects involved in each interaction

Infected males and females reached smaller adult body length than uninfected individuals (Figure 1; males: *t*_72_ **=* *4.29, *p *<* *.001, females: *t*_72_ **=* *4.11, *p *<* *.001). The interaction between female infection status and male infection status explained the variability observed in the number and weight of yolky eggs, reproductive investment, and the number of nymphs (Table 1). Interaction slices revealed that uninfected females produced 43.1% less yolky eggs when crossed with infected than uninfected males (*p *=* *.008; Figure 2a, Table 2). Likewise, when crossed with uninfected males, egg production of infected females decreased 53.4% in comparison with crossings with uninfected females (*p *=* *.006). For yolky egg weight (Figure 2b, Table 2), the only significant slice indicates that crossings between uninfected females × infected males produce eggs 7.3% lighter than eggs from crossings between uninfected females × uninfected males (*p *=* *.041). The reproductive investment (Figure 2c, Table 2) followed a similar but less strong pattern than that observed for the number of yolky eggs, suggesting the overall investment of females in reproduction depends on the infection status of males and females altogether. Regarding nymph number (Figure 2d, Table 2), uninfected females produced 57.7% fewer nymphs when crossed with infected than uninfected males (*p *<* *.001). Likewise, uninfected males sired 36.7% fewer nymphs when crossed with infected than uninfected females (*p *=* *.016). Unexpectedly, infected males sired 42.0% more nymphs when crossed with infected than uninfected females (*p *<* *.001).

**Table 1:** Results of two‐way ANCOVAs and GLM tests for effects of sex and infection status on reproductive dependent variables

Reproductive variable	Female infection status		Male infection status		Female × male infection status	
	Statistics	*p*	Statistics	*p*	Statistics	*p*
Number of yolky eggs	*F*_1,70_ * *=* *2.45	.122	*F*_1,70_ * *=* *0.38	.540	*F*_1,70_ * *=* *4.27	.042
Weight of yolky eggs (mg)	*F*_1,69_ * *=* *0.51	.478	*F*_1,69_ * *=* *0.97	.327	*F*_1,69_ * *=* *4.37	.040
Number of yolkless eggs	*F*_1,70_ * *=* *31.60	<.001	*F*_1,70_ * *=* *0.01	.906	*F*_1,70_ * *=* *1.13	.292
Reproductive investment	*F*_1,69_ * *=* *1.73	.192	*F*_1,69_ * *=* *0.70	.404	*F*_1,69_ * *=* *4.57	.036
E50	*F*_1,70_ * *=* *2.89	.094	*F*_1,70_ * *=* *0.02	.900	*F*_1,70_ * *=* *2.27	.136
Number of nymphs	$\chi_{1}^{2}$ * *=* *46.9	<.001	$\chi_{1}^{2}$ * *=* *104.7	<.001	$\chi_{1}^{2}$ * *=* *84.7	<.001

All analyses included female survival as covariate.

**Table 2:** Statistics for interaction slices between the following: M_0_F_0_–M_0_F_1_, M_0_F_0_–M_1_F_0_, and M_1_F_1_–M_0_F_1_, M_1_F_1_–M_1_F_0_ for reproductive‐dependent variables

Reproductive variable	M_0_F_0_				M_1_F_1_			
	M_0_F_1_		M_1_F_0_		M_0_F_1_		M_1_F_0_	
	Statistics	*p*	Statistics	*p*	Statistics	*p*	Statistics	*p*
Number of yolky eggs	*F*_1,70_ * *=* *8.12	.006	*F*_1,70_ * *=* *3.25	.008	*F*_1,70_ * *=* *1.19	.279	*F*_1,70_ * *=* *0.10	.750
Weight of yolky eggs (mg)	*F*_1,69_ * *=* *1.18	.280	*F*_1,69_ * *=* *4.32	.041	*F*_1,69_ * *=* *0.69	.410	*F*_1,69_ * *=* *3.30	.073
Reproductive investment	*F*_1,69_ * *=* *7.31	.009	*F*_1,69_ * *=* *4.05	.048	*F*_1,69_ * *=* *0.95	.333	*F*_1,69_ * *=* *0.28	.601
Number of nymphs	$\chi_{1}^{2}$ * *=* *5.84	.016	$\chi_{1}^{2}$ * *=* *107.24	<.001	$\chi_{1}^{2}$ * *=* *0.354	.552	$\chi_{1}^{2}$ * *=* *81.74	<.001

M: males, F: females, subscripts 0 and 1 mean uninfected and infected, respectively. *p*‐Values indicate the statistical significance of pairwise‐level effects.

**Figure 3:**
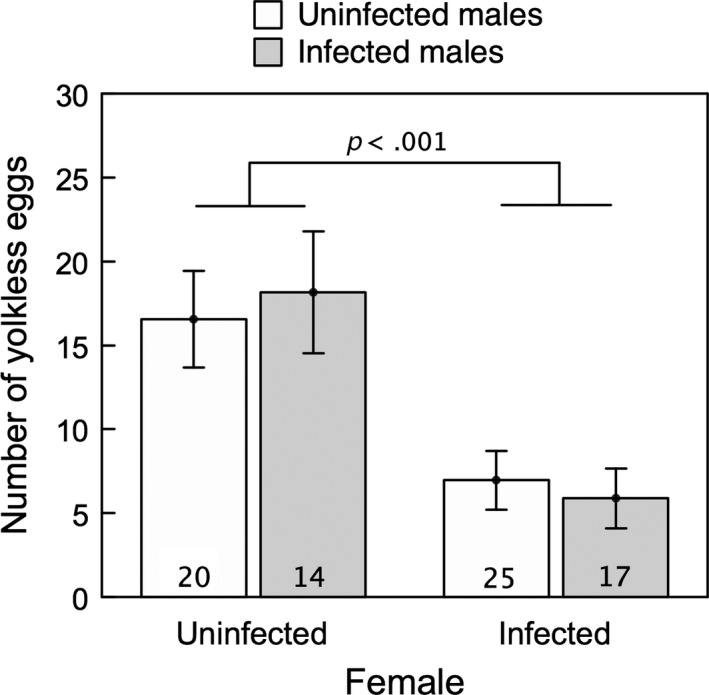
Mean (±1 *SE*) number of yolkless eggs produced by uninfected and infected females when mated with uninfected (white bars) and infected (gray bars) males. Numbers at the bottom of bars indicate the number of mated pairs. *p*‐Value corresponds to the overall female infection status comparison

The interaction does not account for all our results. Only the status of female infection explained the variability observed in the number of yolkless eggs. Infected females produced fewer yolkless eggs than uninfected females, regardless of the infection status of males (Figure 3, Table 1). In addition, the infection did not account for the variability observed in E50 (Table 1).

Uninfected females took larger blood meals than those infected (one‐way ANOVA: *F*_1,72_ **=* *48.45, *p *<* *.001). However, this effect disappeared after including body size as covariate (one‐way ANCOVA: *F*_1,72_ **=* *0.04, *p *=* *.84), indicating that female body size accounted for an important fraction of the variance in the volume of blood ingested. Neither female nor male survival (since the first day of the mating experiment) appeared to be affected by *T. cruzi* infection (*t* test: female: *t*_72_ **= 1.10, *p *=* *.27; male: *t*_72_ **=* *−0.74, *p *=* *.45; Table S1).

## 4 Discussion

The interaction between infection and gender influenced the most reproductive variables (Table 1, Figure 2), implying that the magnitude of parasitism's impact on reproduction in one sex depends on the infection status of the other sex. Parasite‐induced changes in host body size may be the proximal factor involved in host fitness reduction. Previous studies have reported that the protozoan *T. cruzi* has a strong impact on several life history traits of *M. spinolai* (Botto‐Mahan, 2009; Botto‐Mahan, Cattan, & Medel, 2006; Botto‐Mahan et al., 2008). In this study, infected females reached smaller size at maturity compared to those uninfected, suggesting that like other parasite species, *T. cruzi* probably curtails essential nutrients involved in host growth (Hurd, 1990; Thompson, 1983). In this vein, Hurd, Hogg, and Renshaw (1995) suggested that female body size could affect reproduction in two ways. First, fecundity may be limited by the number of ovarioles present in each ovary, which is function of female body size. Second, body weight reduction may negatively affect blood feeding and blood meal utilization for egg production. As in previous studies, *M. spinolai* individuals were exposed to *T. cruzi* from the first nymph stage on, hence increasing the chance of parasite–insect competition. In consequence, it is likely that final insect size results from a trade‐off involving a higher energy allocation to insect survival rather than reproduction (Botto‐Mahan, 2009).

One of the most frequently observed patterns in insect reproduction is size‐assortative mating, that is, the preferential mating between similar sized individuals. Most explanations to this pattern base on mate choice through sexual selection (Arnqvist, 2011; Arnqvist & Rowe, 2005; Baldauf, Kullmann, Schroth, Thünken, & Bakker, 2009; Gagnon & Turgeon, 2011). However, size‐assortative mating may also occur due to mating constraints when males and females differ sufficiently in body size (Crespi, 1989; Han, Jablonski, Kim, & Park, 2010). In the study system, *T. cruzi* reduces the body size of male and female kissing bugs (Figure 1; Botto‐Mahan, 2009). In this way, infected–uninfected pairs had body size mismatches that probably translated into poor physical contact and limited sperm transfer during copulation. Infected–infected pairs, like uninfected–uninfected pairs, may not experience size‐related mating constraints. This may explain (1) why infected males sired more nymphs when crossed with infected females than when crossed with uninfected females (Figure 2d) and (2) why uninfected males sired more nymphs when crossed with uninfected females than they did when crossed with infected females (Figure 2d). The body size hypothesis may also relate to the ability of females to detect and avoid parasites by directly assessing the male infection status through visual, tactile, or olfactory detection, or indirectly through detection of a signal sensitive to infection, such as body size (David & Heeb, 2009). In this study, uninfected females had low egg production and reproductive investment when crossed with infected males (Figure 2). If male body size indicates quality, females lose out by copulating with small‐sized males with high parasite loads, incompatible genotypes, or lacking direct resources to offer.

We have presented evidence that *T. cruzi* influences reproduction through both genders. Even though our study was not designed to inquire into the mechanisms involved in parasite‐induced fitness impact, it is likely that parasites impose a direct cost on female reproduction by reducing resource allocation to reproduction. The effect of *T. cruzi* on males, however, is less clear. There is some evidence for hemipterans that the quantity and quality of the seminal fluid depend on male environment (Kaldun & Otti, 2016). If the seminal fluid composition of *M. spinolai* is altered in the presence of *T. cruzi*, egg number and weight and nymph number may be affected via seminal fluid‐mediated paternal effects (Crean, Adler, & Bonduriansky, 2016; Perry, Sirot, & Wigby, 2013).

The detection of nonadditive effects of gender infection on host reproductive success indicates that studies focusing on only female or male host infection provide a limited view of the effect of parasites on host reproduction. This observation may have important implications to the understanding of size‐assortative mating in insects. A variety of factors have been suggested to cause size‐assortative mating, including mate preferences, mate availability, and mating constraints (Crespi, 1989; Han et al., 2010; Nuismer, Otto, & Blanquart, 2008; Thomas et al., 1995). Results of this study suggest that we should add infection status to this list of variables. The extent to which parasite‐induced size reduction affects size‐assortative mating in natural populations needs to be assessed in future studies.

## Conflict of Interests

We have no conflict of interests.

## Author Contributions

C.B.M. and R.M. designed the study; C.B.M. and V.C. collected data; C.B.M., V.C., and R.M. analyzed data and participated in manuscript writing. All authors gave final approval for publication.

## Data Availability

Raw data are available on the following repository: https://figshare.com/s/5d2e40f59316d1679e1f.
