# Supplementary material for: Sex‐dependent infection causes nonadditive effects on kissing bug fecundity
Source: Ecol Evol. 2017 Apr 9;7(10):3552–7. doi: 10.1002/ece3.2956 (PMC5433981; doi:10.1002/ece3.2956)
Supplement: Supplementary file 1 [file ECE3-7-3552-s001.docx]

Table S1. Summary of descriptive statistics for variables tested for covariates: Volume of ingested blood by females and survivorship. Mean, 1 s.e., sample size (*n*), and range (minimum - maximum values) are shown.

|  |  |  |  | Mean ± 1 s.e. (*n*)  [Range] | | |  |  |  |
| --- | --- | --- | --- | --- | --- | --- | --- | --- | --- |
| Variable | Control males |  | Infected males | |  | Control females | |  | Infected females |
| Volume of ingested blood (mg) | - |  | - | |  | 0.427 ± 0.026 (35)  [0.130-0.826] | |  | 0.314 ± 0.016 (39)  [0.169-0.604] |
| Survivorship (days) | 305.3 ± 19.6 (43)  [84-553] |  | 285.6 ± 22.4 (31)  [36-502] | |  | 254.1 ± 17.2 (35)  [61-473] | |  | 284.2 ± 22.4 (39)  [72-555] |
